# Group Positive Affect and Beyond: An Integrative Review and Future Research Agenda

**DOI:** 10.3390/ijerph17207499

**Published:** 2020-10-15

**Authors:** Jonathan Peñalver, Marisa Salanova, Isabel M. Martínez

**Affiliations:** 1 Department of Education, Universidad Internacional de Valencia, 21, 46002 Valencia, Spain; 2 WANT Research Team, Universitat Jaume I, 12071 Castellón de la Plana, Spain; salanova@uji.es (M.S.); imartine@uji.es (I.M.M.)

**Keywords:** group positive affect, integrative review, antecedents, outcomes, mediators, pitfalls, group performance, happy-productive group

## Abstract

Group positive affect is defined as homogeneous positive affect among group members that emerges when working together. Considering that previous research has shown a significant relationship between group positive affect and a wide variety of group outcomes (e.g., behaviors, wellbeing, and performance), it is crucial to boost our knowledge about this construct in the work context. The main purpose is to review empirical research, to synthesize the findings and to provide research agenda about group positive affect, in order to better understand this construct. Through the PsycNET and Proquest Central databases, an integrative review was conducted to identify articles about group positive affect published between January 1990 and March 2019. A total of 44 articles were included and analyzed. Finding suggests that scholars have been more interested in understanding the outcomes of group positive affect and how to improve the productivity of groups than in knowing what the antecedents are. A summary conclusion is that group positive affect is related to leadership, job demands, job resources, diversity/similarity, group processes, and contextual factors, all of which influence the development of several outcomes and different types of wellbeing at the individual and group levels. However, with specific combinations of other conditions (e.g., group trust, negative affect, and interaction), high levels of group positive affect could cause harmful results. Conclusions shed light on group positive affect research and practice and might help Human Resources professionals to initiate empirically-based strategies related to recruitment, group design and leadership training.

## 1. Introduction

In the words of Barsade and Gibson [1], we are facing an “affective revolution”, due to the growing interest in understanding the role that emotions play in organizations. Although the attention has mainly been placed on individuals [2], literature has increasingly begun to see the relevance of the figure of the group (In this study, we make no distinction between groups and teams, using the two terms interchangeably.) within the organization. Groups contribute to organizational development through their involvement in wellbeing [3], have access to more resources [4], take decisions and solve problems [5], and achieve high levels of performance [6]. Based on substantial empirical evidence, researchers have determined that through several affective linkage mechanisms (e.g., emotional contagion, comparison, empathy) [7], affect not only occurs at the individual level, but also at the group level.

In fact, since Jennifer M. George conducted the first research in 1990 to analyze the positive affective experiences in work teams, a large number of investigations have been carried out (e.g., [8]) and a large number of terms (e.g., group affect, affective climate, and team mood; [9]) have been developed in order to understand this group phenomenon. 

According to George (1990), group affect refers to homogeneous affective reactions among group members. Later, this definition was completed, describing it as affective convergence or the affective composition of the group members [10], resulting from people feeling similar levels of individual emotions when working together [8]. 

Specifically, the positive side of group affect (i.e., group positive affect) has revealed benefits in several group domains, such as behaviors, resources, wellbeing, and performance. For example, group positive affect build social interactions among members by developing others’ ideas, improving communication, and encouraging bonds. [11,12]. Moreover, group positive affect motivates groups to explore fitting behaviors for group performance, such as sharing goals, coordinating the activities, and feeding a positive work climate [13]. Based on the Broaden-and-Build Theory [14], which states that positive affect create resourceful environments and promote wellbeing, Salanova and colleagues [15,16] have consistently confirmed that the groups experience wellbeing (i.e., resilience and engagement) due to feeling positive affect. Moreover, from a leadership perspective, group positive affect has been considered as psychosocial mechanisms that could explain this relationship between leader and group performance [17]. Therefore, the aforementioned benefits emphasize the importance of developing group positive affect to work in healthy, resilient, and productive organizations [18]. 

The interest in group positive affect has produced considerable growth in the research, making it necessary to constantly review the state-of-the-art in order to establish the foundations for the future research agenda. To date, multiple reviews on the topic have been conducted [8,9,19,20,21,22,23,24,25,26]. However, the aforementioned reviews present some limitation that we would like to overcome. (1) Reviews are based on narrative review. As Pae [27] noted, narrative reviews present several limitations, such as not predefining the protocol during the search stage or including studies for review based on authors’ hunches and research knowledge. Consequently, we propose to conduct an integrative review considered as, “the broadest type of research review methods allowing for the simultaneous inclusion of experimental and non-experimental research in order to more fully understand a phenomenon of concern” ([28], p. 547). Moreover, it guarantees a rigorous process of identification, analysis, and synthesis of the results, without the need to focus on one specific question [29]. According to Cronin and George [30], an integrative review leads to redirect research on the topic through the synthesis and critique of knowledge. (2) Reviews present a narrow focus of construct. That is, reviews examine the relationship between group positive affect and some variables. For instance, Knight and Eisenkraft [23] performed the first meta-analysis exploring the mean effect of group positive affect on social integration and group performance. However, Knight and Eisenkraft [23] only focused on two specific outcomes (i.e., social integration and group performance), leaving out many antecedents and outcomes that would make it possible to obtain a comprehensive view of group positive affect. Moreover, with the exception of Ashkanasy and Humphrey [19], previous reviews have shown a lack of attention to the relationship between group positive affect (group level) and variables of different levels (i.e., individual and organizational). Thus, we approach the study of group positive affect from a multilevel perspective that encompasses the construct within a complete process (i.e., antecedents, outcomes, mediators, and moderators). 

To fill this research gap, the purpose of the current integrative review is to (1) critically review empirical research about positive affective experiences at the group level of analysis, and (2) synthesize the findings in order to advance the understanding of group positive affect, and (3) provide a wide-ranging research agenda of group positive affect. To structure the process of review and synthesize the findings, five broad research questions guided this work

**Research Question1**: *How is group positive affect operationalized*?

**Research Question2**: *What are the antecedents of group positive affect*?

**Research Question3**: *What are the outcomes of group positive affect*?

**Research Question4**: *Between what variables do group positive affect works as a psychosocial mechanism*?

**Research Question5**: *Under what circumstances do high levels of group positive affect lead to negative outcomes*?

## 2. Method

Considering the characteristics of integrative review (i.e., inclusion of experimental and non-experimental research, no need to focus on one specific question, and search process clearly documented), this type of review was chosen. Based on Whittemore and Knafl’s guidelines [28], the integrative review was implemented in five stages: research question identification, literature search, search outcome, data synthesis, and presentation of results.

### 2.1. Literature Search 

First, an electronic search was carried out of literature published prior to March 2019 using the following databases: PsycNET and Proquest Central. In order to identify relevant studies, through the recent reviews, we checked the different terms referring to positive affective experiences at the group level. A keyword search was conducted with a set of keywords: (group OR team OR collective OR workgroup) AND (affective climate OR affect OR mood OR emotion OR trait OR tone) AND positive.

Second, in parallel, tracking down the references cited by relevant sources, we performed a manual search. Manual search is considered a useful complement because articles might be inaccurately indexed or might fail to include keywords during the literature search [31]. 

Five inclusion criteria were considered: (1) The study had to be empirical (i.e., experimental and non-experimental studies); (2) The study had to be published in English or Spanish in a scientific peer-review journal between January 1990 and March 2019; (3) Group positive affect had to be operationalized as positive affect that emerges among group members, not as an affective linkage mechanism (e.g., emotional contagion) or similar affective construct (e.g., affective presence); (4) Group positive affect had to be evaluated in a work context such as a laboratory (e.g., organizational simulation or task decision) or field (e.g., organization); and (5) Agreement (e.g., AD, [32]) or reliability (e.g., ICC1, ICC2, [33]) indices had to be calculated in order to statistically justify the aggregation of group positive affect at the group level of analysis. According to Bliese [33], for theoretical and practical reasons, aggregated constructs require evaluating these indices to provide construct validity in order to identify emerging phenomena.

### 2.2. Search Outcome

All the articles that contained the keywords were incorporated, as well as articles found through relevant sources. Using the inclusion criteria, the articles were selected. First, the title and abstract were reviewed, and then the full text. During the process, articles were discussed through peer review. Moreover, extra information was requested to the authors´ articles.

**Figure 1:**
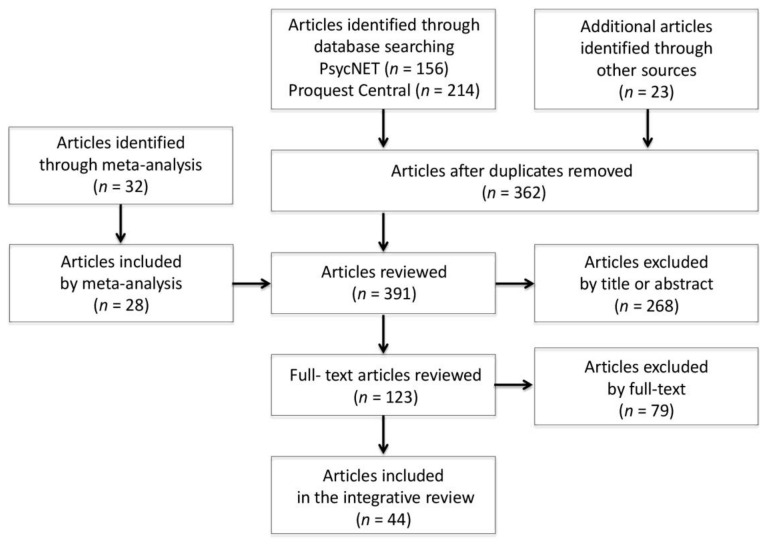
Flow diagram clarifying the literature search and selection process.

Two issues were detected in this stage: (1) Knight and Eisenkraft meta-analysis [23] was detected in the database. The articles considered in the aforementioned meta-analysis were reviewed in order to determine whether they could be included in the present review. Studies considered grey literature (e.g., doctoral dissertations and chapters) were searched to find out whether the authors had published similar results on the topic; (2) Although some reviewed articles did not meet the third inclusion criterion [34,35], they were included in the database because the authors made arguments in the article that this calculation was not necessary. The solutions adopted involve an effort to guarantee the quality of the integrative review. Figure 1 clarifies the literature search and article selection process.

### 2.3. Data Synthesis

First, following Cooper´s recommendations [36], we analyzed methodological characteristics such as the group sample, Cronbach’s alpha, and response rate, in order to evaluate the quality of the research. In addition, in accordance with multilevel theory, we analyzed: (1) Referent-Shift Consensus [37], meaning that there is a shift in the referent (i.e., “My team feels…), as opposed to Direct Consensus (i.e., “I feel….”); (2) Fuzzy composition processes [33] to statistically demonstrate agreement and reliability. Other methodological details considered are shown in Table 1.

Further, Table 2 contains information on the articles´ authors and years of publication, term used to refer to group positive affect, instrument, sample, major variables (i.e., independent variable, mediator variable, moderator variable, and dependent variable). Results are structured in order to answer the five research questions aforementioned. 

## 3. Results

We used Mendeley to store, organize, and read the 46 quantitative studies analyzed. Specifically, 44 articles were accepted, but articles nos. 6 and 28 were composed of two studies each one of them (for this reason, from now, we will refer to studies and not to articles). 

As the majority of the studies in our review were cross-sectional, it is impossible to make causal inferences about these variables. However, we use the terms antecedents and outcomes to refer to independent and dependent variables, respectively.

### 3.1. Research Question 1. How is Group Positive Affect Operationalized?

The methodological characteristics of all the articles and studies examined are displayed in Table 1. The data show that the number of groups ranged between 19 and 417, while the number of members per group ranged between from 2 to 38. The instruments used to measure group positive affect showed Cronbach’s alpha values between 0.70 and 0.96. The study designs were primarily field studies (34 studies), whereas 12 were carried out in a laboratory; 31 studies were cross-sectional, and 15 were longitudinal. Regarding the referent in the scale, 16 studies used Referent Shift Consensus and 30 used Direct Consensus. In order to evaluate agreement, the Rwg index was the most commonly used (33 studies, values of between 0.49 and 0.95), followed by the AD index (7 studies, values of between 0.10 and 0.67), whereas the reliability values ranged between 0.08 and 0.97 for ICC1, and between 0.19 and 0.86 for ICC2. Participant response rates ranged between 11.8% and 98% (18 studies did not reported). With regard to cross-level relations, most of the studies analyzed (39 studied) focused on establishing relationships at the group level. Only seven studies established cross-level relationships between different levels of analysis: six group-individual level and one group-organizational level. 

The studies included in the integrative review, we noted that, in all, twenty-two different terms were used to refer to positive affective experiences in groups. However, the term used the most was positive group affective tone (eight studies), followed by positive affective tone (seven studies), group positive affect (five studies), and positive affect (three studies). 

With regard to measurement instruments, the Positive and Negative Affect Schedule (PANAS, [38]) was used the most (18 studies); 6 studies used HERO [39]; 4 studies used the Job Affect Scale (JAS, [40]); 4 studies used scales based on the Affective Circumplex Model (e.g., [41]); 3 studies used the Affective Well-being Scale [42]; 2 studies used the Job-Related Affective Well-Being Scale (JAWS, [43]); and 6 used other scales (e.g., self-constructed, unavailable data).

**Table 1:** Methodological characteristics of the included studies.

	Source	*n* (Groups)	Group Size: Range; M (DT)	Cronbach α Instrument	Design	Composition Model	Agreement	Reliability	Response Rate	Statistical Analysis	Unit of Analysis
**1**	Bashshur et al. (2011) [44]	152–179	4.63 (1.84)	0.96	Field. LG	DC	AD = 0.54	ICC1 = 0.23, ICC2 = 0.60	79.73–90.12%	Polynomial regression	Group
**2**	Bramesfeld & Gasper (2008) [45]	30	3	0.94	Lab. CS	DC	Rwg = 0.75	UD	UD	ANOVA, Mediation analyses	Group
**3**	Bustamante et al. (2014) [46]	264	5 (1.54)	0.92	Field. CS	RSC	UD	ICC1 = 0.29, ICC2 = 0.62	UD	SEM	Group
**4**	Chi, & Huang (2014) [47]	61	4.57 (2.52)	0.93	Field. CS	DC	Rwg = 0.95	ICC1 = 0.21, ICC2 = 0.58	76%	SEM	Group
**5**	Chi, et al. (2011) [17]	85	7.34 (2.80)	0.89	Field. CS	DC	Rwg = 0.91	ICC1 = 0.23	69%	SEM	Group
**6**	Collins et al. (2015) [48]	Study 1: 61	Study 1: 3 to 7; 3.59 (.93)	0.90–0.91	Lab. LG	DC	Rwg = 0.78	ICC1 = 0.12, ICC2 = 0.31	86.05%	Hierarchical regression	Group
		Study 2: 47	Study 2: 3 to 4; 2.64 (.61)	0.89–0.91	Lab. LG	DC	Rwg = 0.88	ICC1 = 0.23, ICC2 = 0.44	41.89%		Group
**7**	Dimotakis et al. (2012) [49]	21	5	0.94	Lab. LG	DC	Rwg = 0.61–0.72	ICC1 = 0.20, ICC2 = 0.84	UD	Hierarchical regression	Group
**8**	Gamero et al. (2008) [50]	156	4 to 14; 5.83 (1.89)	0.95	Field. LG	DC	AD = 0.55–0.58	ICC1 = 0.19, ICC2 = 0.51–0.52	87.7–95.1%	Hierarchical regression	Group
**9**	George (1990) [51]	26	2 to 16	0.80	Field. LG	DC	UD	ICC1 = 0.87	84.67%	Regression	Group
**10**	George (1995) [52]	41	4 to 9	0.91	Field. CS	DC	UD	ICC1 = 0.88	72%	Regression	Group
**11**	Gil et al. (2015) [53]	110	6.28 (4.4)	0.92	Field. CS	RSC	UD	ICC1 = 0.13	UD	Regression	Group
**12**	González-Romá ,& Gamero (2012) [54]	59	3 to 9; 4.39 (1.39)	0.92	Field. LG	DC	AD = 0.47	UD	95.3–98%	Regression	Group
**13**	Hentschel et al. (2013) [55]	38	3 to 19; 8 (4.64)	0.85	Field. CS	RSC	Rwg = 0.92	ICC1 = 0.44, ICC2 = 0.86	69.13%	Hierarchical regression	Group
**14**	Hmieleski et al. (2011) [34]	179	51	0.91	Field. LG	RSC	Rwg = 0.81–0.72	UD	11.8%	Hierarchical regression, bootstrapping	Group, organization
**15**	Kim et al. (2016) [56]	50	UD	0.86	Field. CS	RSC	Rwg = 0.84	ICC1 = 0.12, ICC2 = 0.44	82%	Hierarchical regression	Group, individual
**16**	Kim, & Shin (2015) [57]	97	6.1 (2.1)	0.84	Field. CS	DC	Rwg = 0.85	ICC1 = 0.15, ICC2 = 0.47	80%	Hierarchical regression	Group
**17**	Kim et al. (2013) [58]	42	3 to 15; 6.21 (3)	0.87	Field. CS	DC	Rwg = 0.93	ICC1 = 0.19, ICC2 = 0.63	74%	HLM	Group, individual
**18**	Klep et al. (2011) [59]	70	3	0.93	Lab. CS	DC	Rwg = 0.86	ICC1 = 0.54, ICC3 = 0.97	UD	ANOVA	Group
**19**	Knight (2015) [60]	33	10 to 17; 11.54 (1.33)	UD	Field. LG	RSC	Rwg = 0.90–0.92	ICC1 = 0.08–0.09, ICC2 = 0.43–0.47	74–94%	Growth models, regression	Group
**20**	Lee et al. (2016) [61]	100	3 to 17	0.83	Field. LG	RSC	Rwg = 0.91	ICC1 = 0.32, ICC2 = 0.69	UD	Regression	Group
**21**	Levecque, et al. (2014) [62]	97	UD	0.81	Field. CS	DC	AD = 0.67, Rwg = 0.84	ICC1 = 0.24, ICC2 = 0.70	81.6%	Hierarchical logistic regression	Group, individual
**22**	Lin et al. (2014) [63]	47	6.5	0.88	Field. CS	DC	Rwg = 0.95	ICC1 = 0.25, ICC2 = 0.59	63.1%	Hierarchical regression	Group
**23**	Mason (2006) [64]	24	3 to 25; 7.66 (5.06)	0.83	Field. CS	DC	Rwg = 0.79	ICC1 = 0.09	>75%	Semipartial correlations	Group
**24**	Mason, & Griffin (2003) [65]	97	3 to 30; 15.58 (7.80)	0.88–0.89	Field. LG	RSC	Rwg = 0.85	ICC1 = 0.21–0.22, ICC2 = 0.59–0.69	73%	HLM	Group, individual
**25**	Mason, & Griffin (2005) [66]	55–66	3 to 30; 9.32	UD	Field .CS	RSC	Rwg = 0.63	UD	66.5%	Hierarchical regression	Group
**26**	Meneghel, et al. et al. (2014) [15]	216	2 to 38; 4.99 (4.20)	UD	Field. CS	RSC	AD = 0.10–0.14	ICC1 = 0.72–0.97	UD	SEM	Group
**27**	Paulsen et al. (2016) [67]	34	UD	0.75–0.92	Lab. LG	DC	Rwg = 0.78	UD	UD	MSEM	Group
**28**	Peñalver et al. (2019) [13]	Study 1: 112	Study 1: 2 to 5	0.93	Lab. CS	RSC	AD = 0.54–0.59	ICC1 = 0.10–0.18	UD	SEM	Group
		Study 2: 417	Study 2: 2 to 35; 5.14 (4.4)	0.93	Field. CS	RSC	AD = 0.92–0.94	ICC1 = 0.13–0.16	UD		Group
**29**	Rego et al. (2014) [35]	106	12.2 (6.89)	0.71	Field. CS	RSC	UD	UD	66%	Path analysis approach	Group
**30**	Salanova et al. (2011) [16]	19	4 to 7	0.70–0.85	Lab. LG	RSC	Rwg = 0.84–0.89	UD	UD	SEM	Group
**31**	Sánchez-Cardona et al. (2018) [68]	130	2 to 18; 5	0.89	Field. CS	RSC	Rwg = 0.75	ICC1 = 0.33, ICC2 = 0.68	UD	SEM	Group
**32**	Seong & Choi (2014) [69]	96	3 to 21; 10.35 (4.91)	0.96	Field. CS	RSC	Rwg = 0.94	ICC1 = 0.11, ICC2 = .53	85.7%	SEM	Group
**33**	Shin (2014) [70]	98	4 to 11; 5.8 (2.4)	0.88	Field. CS	DC	Rwg = 0.84	ICC1 = 0.19, ICC2 = 0.58	72%	SEM	Group
**34**	Shin et al. (2019) [71]	116	3 to 11; 5.58 (2.2)	0.95	Field. CS	DC	Rwg = 0.94	ICC1 = 0.11, ICC2 = 0.45	68%	HLM	Group
**35**	Sy and Choi (2013) [72]	102	3 to 5	UD	Lab. LG	DC	Rwg = 0.49–0.84	ICC1 = 0.29–0.55, ICC2 = 0.65–0.88	UD	Hierarchical regression	Group
**36**	Tang, & Naumann (2016) [73]	47	UD	UD	Field. CS	DC	Rwg = 0.90	UD	60.3%	HLM	Group
**37**	Tangue et al. (2010) [74]	71	2 to 4	0.71	Field. CS	DC	Rwg = 0.89	ICC1 = 0.09, ICC2 = 0.19	UD	Hierarchical regression	Group
**38**	Teng, & Luo (2014) [75]	123	2 to 5	0.74	Field. CS	DC	Rwg = 0.71–0.99	UD	96.1%	SEM	Group
**39**	Tran et al. (2012) [76]	20	4 to 8; 5.3	UD	Lab. LG	DC	IRR = 0.95–0.98	ICC = 0.12–0.46	UD	Correlations, non-parametric test	Group
**40**	Tsai et al. (2011) [77]	68	5.9 (2.5)	0.88	Field. CS	DC	Rwg = 0.92–0.95	ICC1 = 0.13, ICC2 = 0.45	71%	HLM	Group
**41**	Tu (2009) [78]	106	3 to 9; 5.71	0.92	Field. CS	DC	Rwg =0.92	ICC1 = 0.33, ICC2 = 0.78	17.2%	HLM	Group
**42**	Van Knippenberg et al. (2010) [79]	178	3	0.89	Lab. CS	DC	Awg = 0.19	UD	UD	Regression	Group
**43**	Volmer (2012) [80]	21	3	0.88	Lab. CS	DC	Rwg = 0.72	UD	UD	HLM	Group, individual
**44**	Zhang et al. (2017) [81]	74	4.39	0.88	Field. CS	DC	Rwg = 0.88	ICC1 = 0.26, ICC2 = 0.68	UD	HLM	Group, individual

Note: UD (unavailable data); LG (Longitudinal study); CS (Cross-sectional study); DC (Direct Consensus); RS (Referent Shift); SEM (Structural Equation Modelling); HLM (Hierarchical lineal modelling); MSEM (Multilevel structural equation modelling).

**Table 2:** Summary of studies included in the review.

	Source	Term	Instrument	Sample	Independent Variable	Moderator Variable	Mediator Variable	Dependent Variable	Informant (Variable)	Country	Journal
**1**	Bashshur et al. (2011) [44]	Team positive affect	Affective Well-being Scale [42]	Employees in different branches of three savings banks in the same geographical region	Team climate, Manager perception of team climate			Group positive affect	Managers (Team climate)	Spain	Applied Psychology
**2**	Bramesfeld & Gasper (2008) [45]	Happy mood	UD	Students from a course	Mood manipulation (e.g., Group positive affect), Evidence distribution		Focus on the evidence	Group performance	Objective (Group performance)	U.S.A	Universitas Psychologica
**3**	Bustamante et al. (2014) [46]	Positive emotions	HERO [39]	Employees from service sector	Empathy		Positive emotions	Quality of service	Managers (Quality of service)	Spain	Revista Latinoamericana de Psicología Positiva
**4**	Chi & Huang (2014) [47]	Positive group affective tone	Positive and Negative Affect Schedule [38]	Research and development (R&D) teams from high-technology firms	Transformational leadership		Team learning goal orientation, Team avoiding goal orientation, Group positive affect, Negative group affective tone.	Team performance	Managers (Team performance)	Taiwan	Group & Organization Management
**5**	Chi et al. (2011) [17]	Positive group affective tone	Positive and Negative Affect Schedule [38]	Sales teams from five insurance firms	Leader positive moods		Group positive affect, Transformational Leadership, Team goal commitment, Team satisfaction, Team helping behaviors.	Team performance	Leaders (Leader positive moods, Team performance), Organizational database (Team performance)	Taiwan	Small Group Research
**6**	Collins et al. (2015) [48]	Positive affective tone (Study 1)	Positive and Negative Affect Schedule [38]	University students completing a business communication course	Group positive affect	Management of others’ emotions.		Team improvement; Team task	Objective (Team improvement, Team task)	Australia	Organizational Behavior and Human Decision Processes
		Positive affective tone (Study 2)	Positive and Negative Affect Schedule [38]	University students frombusiness course	Group positive affect	Management of others’ emotions		Team performance	Objective (Team performance)		The Journal of Creative Behavior
**7**	Dimotakis et al. (2012) [49]	Positive affect	Positive and Negative Affect Schedule [38]	University students	Regulatory focus, Team structure, Task characteristics	Team structure	Helping behaviors, Group positive affect	Task performance, Task satisfaction		U.S.A	Journal of Management & Organization
**8**	Gamero et al. (2008) [50]	Affective climate. Enthusiasm climate	Affective Well-being Scale [42]	Employees from saving banks	Task Conflict T1, Group positive affect T1		Relationship conflict T2	Group positive affect T2		Spain	British Journal of Management
**9**	George (1990) [51]	Positive affective tone of the work group	Job Affect Scale [40]	Salespeople working for a large department store	Negative affective tone, Group positive affect, Commission			Prosocial Behavior, Absence	Organization (Absenteeism)	U.S.A	The Asia-Pacific Education Researcher
**10**	George (1995) [52]	Group positive affective tone	Modified Positive and Negative Affect Schedule [38]	Salespeople from a retail organization	Leader positive mood, Group positive affect			Group performance	Sales manager (group performance, leader positive mood)	U.S.A	Revista de Psicología Del Trabajo y de Las Organizaciones
**11**	Gil et al. (2015) [53]	Positive affect in work teams	HERO [39]	Employees from service organizations	Work team size, Economic sector, Gender, Type of contract, Organizational tenure			Group positive affect		Spain	Journal of Organizational Behavior
**12**	González-Romá & Gamero (2012) [54]	Positive team mood	Affective Well-being Scale [42]	Branches from a saving bank	Support climate		Group positive affect	Team members’ perceived team performance, Managers’ team effectiveness ratings	Branch Manager (team performance)	Spain	Industrial Marketing Management
**13**	Hentschel et al. (2013) [55]	Positive team affective tone	Job-Related Affective Well-Being Scale [43]	Different sectors (e.g., manufacturing, and technological, administration, medical)	Perceived diversity	Diversity beliefs	Group positive affect, Negative team affective tone	Team identification, Relationship conflict		Germany	Organization Science
**14**	Hmieleski et al. (2011) [34]	Positive team affective tone	Job-Related Affective Well-Being Scale [43]	CEOs of top management teams from new firms	Shared authentic leadership		Group positive affect	Firm performance	CEOs (Shared authentic leadership, Group positive affect), Dun and Bradstreet database (Firm performance)	U.S.A	Administrative Sciences
**15**	Kim et al. (2016) [56]	Positive affective climate	Affective Circumplex [82]	Employees with different job position	Positive trait affect, Negative trait affect, Group positive affect, Group reflexivity	Group positive affect, Group reflexivity		Employee creativity	Supervisor (employee creativity)	Korea	Social Behavior and Personality: An International Journal
**16**	Kim & Shin (2015) [57]	Group positive affect	Positive and Negative Affect Schedule [38]	Employee from different size and sector organizations	Cooperative group norms, Group positive affect		Collective efficacy	Team creativity	Team leader (team creativity)	Korea	Applied Psychology
**17**	Kim et al. (2013) [58]	Group trait positive affect	Positive and Negative Affect Schedule [38]	Office workers across different industries (telemarketing, financial, pharmaceutical, and media industries)	Individual trait positive affect	Group positive affect, Group positive affect diversity		Commitment, Job satisfaction, OCB		Korea	Universitas Psychologica
**18**	Klep et al. (2011) [59]	Positive mood	Self-constructed	Dutch University students	Manipulation work group mood (e.g., Group positive affect), Interactive affectivesharing			Work group performance, Group belongingness, Group information sharing	Observers (Group belongingness, Group information sharing), Objective (Work group performance)	Netherlands	Group & Organization Management
**19**	Knight (2015) [60]	Team positive mood	Circumplex model of affect [41]	Members from a military academy	Group positive affect, Time	Team exploratory search		Team exploratory search, Team performance	Objective (Team performance)	U.S.A	Small Group Research
**20**	Lee et al. (2016) [61]	Group positive affect	Positive and Negative Affect Schedule [38]	Employees in a manufacturing plant from China	Past group performance, Group Vicarious learning, Group social persuasion, Group positive affect	Group Trust	Group efficacy	Group Performance	Organization (group performance)	China	Organizational Behavior and Human Decision Processes
**21**	Levecque et al. (2014) [62]	Affective team climate	UD	Workers in the Volvo Car plant in Ghent, Belgium	Group positive affect, Job demands, Perceived team climate, Job control, Social support	Group positive affect, Perceived team climate, Job control, Social support		Psychological distress		Belgium	The Journal of Creative Behavior
**22**	Lin et al. (2014) [63]	Positive group affect	Positive and Negative Affect Schedule [38]	MBA alumni for the most recent three years from a local university	Group positive affect, Negative group affect		Group efficacy	Group identification		Taiwan	Journal of Management & Organization
**23**	Mason (2006) [64]	Positive affect	Job Affect Scale [40]	This sample was diverse and there was wide range in the type of tasks performed by each work group, ranging from patient care (in a hospital) to client service (in a call center) to replenishment of stock (on a factory floor) to management (within a fast-food chain).	Group time, Task variety, Outcome interdependence, Heterogeneity in backgrounds, Gender Diversity, Age Diversity, Communication quality, Cohesion, Task interdependence, Frequency of meetings			Group positive affect		Australia	British Journal of Management
**24**	Mason & Griffin (2003) [65]	Positive affective tone	Queensland Public Agency Staff Survey [83]	Workers for an Australian state government agency	Group positive affect			Group absenteeism	Organization (Absenteeism)	Australia	The Asia-Pacific Education Researcher
**25**	Mason & Griffin (2005) [66]	Positive affective tone	Job Affect Scale [40]	Employees from a variety of different industries operating within both the public and private sector, and the functions of the work groups varied widely, from management to customer service to the replenishment of stock on a factory floor	Group task satisfaction, aggregated individual job satisfaction, Group positive affect, Negative affective tone			Civic helping (group and supervisor), Performance (supervisor), Sportsmanship (group and supervisor), Absenteeism norms (group and supervisor)	Supervisor (Performance, Sportsmanship, Civic helping)	Australia	Revista de Psicología Del Trabajo y de Las Organizaciones
**26**	Meneghel et al. (2014) [15]	Collective positive emotions	HERO [39]	Employees from service, industry and construction sector in Spain	Group positive affect		Team resilience	Team in role performance, Team extra-role performance	Supervisor (Team in role performance, Team extra-role performance)	Spain	Journal of Organizational Behavior
**27**	Paulsen et al. (2016) [67]	Positive group affective tone	Short form of Positive and Negative Affect Schedule [38]	Students from a software engineering course at a German university	Group positive affect, Negative group affective tone, Project phase	Project phase		Team performance (experts), Team performance (self-rated)	Experts (Team performance)	Germany	Industrial Marketing Management
**28**	Peñalver et al. (2019) [13]	Group positive affect (Study 1)	HERO [39]	University students, full time workers from a wide range of occupations and others	Group positive affect		Group social resources	In- performance extra-role performance, and creative performance	In- performance extra-role performance (leader), and creative performance (external evaluators)	Spain	Organization Science
		Group positive affect (Study 1)	HERO [39]	Employee from different size and sector organizations	Group positive affect		Group social resources	In- performance extra-role performance	In- performance extra-role performance (supervisor)	Spain	Administrative Sciences
**29**	Rego et al. (2014) [35]	Positive affective tone	Positive affective tone [84]	Brazilian retail organization	Group positive affect	Negative affective tone	Store creativity	Store performance	Supervisor (Group positive affect, Store creativity), Organization (Store performance)	Portugal	Social Behavior and Personality: An International Journal
**30**	Salanova et al. (2011) [16]	Collective positive affect	Enthusiasm-depression scale [85,86]	University students	Efficacy beliefs		Group positive affect	Engagement		Spain	Applied Psychology
**31**	Sánchez-Cardona et al. (2018) [68]	Team positive affect	HERO [39]	Employee from different size and sector organizations	Leader intellectual stimulation		Group positive affect	Team learning		Spain	Universitas Psychologica
**32**	Seong & Choi (2014) [69]	Group positive affect	Circumplex Model of Affect. [87]	Korean company in the defense industry	Leader positive affect		Group positive affect, Group-level goal fit, Group-level ability fit, Relationship conflict, Task conflict	Group performance	Supervisor (Relationship conflict, Task conflict, Group performance)	Korea	Group & Organization Management
**33**	Shin (2014) [70]	Positive group affective tone	Positive and Negative Affect Schedule [38]	Teams varied in functional areas (e.g., planning and strategy, sales, human resource management and development, research and development, finance and accounting, and marketing) from different organizations	Group positive affect, Negative group affective tone		Team reflexivity, Team promotion focus, Team prevention focus	Team creativity	Leaders (Team creativity)	UD	Small Group Research
**34**	Shin et al. (2019) [71]	Positive group affective tone	Positive and Negative Affect Schedule [38]	Full-time employees from 17 companies in South Korea, representingdiverse firm sizes and industries	Group positive affect	Team leader transformational leadership	Team reflexivity	Team creativity performance, Team change organizational citizenship behavior	Team leader (Team creativity performance, Team change organizational citizenship behavior)	South Korea	The Journal of Creative Behavior
**35**	Sy & Choi (2013) [72]	Positive group mood convergence	Job Affect Scale [40]	Students from management courses	Group-Leader affective diversity, Member affective diversity, Mood induction in leaders	Interpersonal attraction toward leader, Interpersonal attraction toward group, Emotional contagion susceptibility	Group positive affect, Negative group mood convergence	Group positive affect, Negative group mood convergence	Leader (Affective diversity, Interpersonal attraction, mood)	U.S.A	Organizational Behavior and Human Decision Processes
**36**	Tang & Naumann (2016) [73]	Team positive mood	Positive and Negative Affect Schedule [38]	Employees in research institutes in China (basic research, high technology R&D, other fields)	Work value diversity	Group positive affect	Knowledge sharing	Team creativity		U.S.A	Journal of Management & Organization
**37**	Tangue et al. (2010) [74]	Positive group affective tone	Circumplex model of affect [41]	Employees from commercially oriented service organizations, such as shops, bars, restaurants, and physiotherapists’ offices,	Group positive affect, Negative group affective tone	Group identification.		Willingnessto engage in OCB, Perceived team performance			British Journal of Management
**38**	Teng & Luo (2014) [75]	Group affective tone	Positive and Negative Affect Schedule [38]	College students studying hospitality and tourism management.	Perceived social loafing, Perceived social interdependence		Group positive affect	Group productivity, Group final grades	Lecturer (group final grades)	Taiwan	The Asia-Pacific Education Researcher
**39**	Tran et al. (2012) [79]	Achievement emotions, Approach emotions	Emotion Wheel [88]	Managers taking part in executive development seminars	Group positive affect, Positive ratio			Alternative generation, Alternative evaluation		France	Revista de Psicología Del Trabajo y de Las Organizaciones
**40**	Tsai et al. (2011) [77]	Positive Group Affective Tone	Positive and Negative Affect Schedule [38]	R&D teams from high-technology firms	Group positive affect	Negative Group Affective Tone, Team trust		Team creativity	Leaders (Team creativity)	Taiwan	Journal of Organizational Behavior
**41**	Tu (2009) [78]	Positive affective tone	Positive and Negative Affect Schedule [38]	New product development teams of high-technology firms from the Taiwan Stock Exchange	Group positive affect, Negative affective tone	Organizational support, Organizational control		Team creativity	Supervisor (team creativity)	Taiwan	Industrial Marketing Management
**42**	Van Knippenberg et al. (2010) [79]	Positive mood	UD	University students	Manipulation mood (e.g., Group positive affect)	Trait negative affect	Information elaboration	Decision quality, Information elaboration	Audio-video records (Information elaboration), Objective (Decisión quality)	Netherlands	Organization Science
**43**	Volmer (2012) [80]	Group affective tone	UWIST mood adjective checklist [89]	University students	Manipulation of Leader´s mood		Group positive affect	Team Performance, Team potency, Team goal commitment, Individual Mood		Germany	Administrative Sciences
**44**	Zhang et al. (2017) [81]	Positive group affective tone	Positive and Negative Affect Schedule [38]	Research and development groups employed by high-technology companies located in China	Leader´s psychological capital, Group positive affect	Leader´s psychological capital	Core self-evaluation	Work engagement	Leaders (Leader´s psychological capital)	China	Social Behavior and Personality: An International Journal

Note: UD (unavailable data).

### 3.2. Research Question 2. What are the Antecedents^2^ of Group Positive Affect?

Five studies reported antecedents of group positive affect. Although the antecedents studied were varied, we have classified them in two categories.

*Group processes*: Congruent with previous studies at the individual level about how disagreement on task issues is associated with relationship conflicts and employee wellbeing, Gamero, González-Romá, and Peiró [50] proposed a homologous model showing that relationship conflict (T1) fully mediates the relationship between task conflict (T2) and group positive affect (T2). In other words, through a process of biased information, criticism, and debate during tasks, groups could unknowingly unleash relationship conflict and reduce the chances of working in a positive and enthusiastic environment. With regard to biases in companies, Bashshur, Hernández, and González-Romá [44] addressed the importance of organizational support climate agreement through two steps: (1) Team climate for organizational support has a positive impact on group positive affect over time; (2) Differences in team and manager perceptions of team climate produce detrimental effects on group positive affect, whereas their agreement boosts group positive affect when both the team and manager perceive high levels of team climate. Moreover, Mason [64] suggested a series of predictors of group positive affect by means of semi partial correlations. Results showed that the frequency of team meetings was most positively related to group positive affect, followed by the time spent performing tasks for which the team is responsible. 

*Contextual factors*: Based on social identity theory [90], Gil, Llorens and Torrente [53] focused on examining the shared characteristics that are related to shared positive affect among group members. Controlling for team size and economic sector, a similar type of contract and organizational tenure were positively related to group positive affect. That is, in order for group positive affect to emerge, members should perceive themselves as equals and have a greater sense of affiliation with the group. On the other hand, Sy and Choi [72] developed and tested a theoretical framework to explain the process through which personality diversity (i.e., leader–group as GLAD, member–member as MAD) produces modifications in group positive affect over time, as well as the social variables (i.e., interpersonal attraction and emotional contagion susceptibility) that participate in this process. Findings revealed that at the beginning (second data collection), MAD, GLAD, and leader attraction were significantly related to group positive affect, MAD and GLAD negatively and leader attraction positively. In fact, the effect of GLAD was moderated by both emotional contagion susceptibility and leader attraction. Thus, when high levels of emotional contagion susceptibility are present, the levels of diversity between the leader and the group (i.e., high or low diversity) imply greater change in group positive affect. In other words, high emotional contagion susceptibility and high leader-group diversity implies low levels of group positive affect. However, high emotional contagion susceptibility and low leader-group diversity implies high levels of group positive affect. With regard to leader attraction, when groups present high levels of interpersonal attraction to the leader, they display minimal differences in group positive affect, regardless of the levels of diversity between the leader and the group. In the third data collection, data showed that only MAD continued to be negative and significant; that is, the effect of leader diversity was lost in the long term. Specifically, the effect of MAD was moderated by the group member attraction. When groups present high levels of members’ interpersonal attraction, the levels of diversity among the group members completely determine the group positive affect, so that high diversity means lower levels of group positive affect, and, on the contrary, less diversity means higher levels of group positive affect. Briefly, in all circumstances, personality diversity hinders the development of group positive affect.

### 3.3. Research Question 3. What are the Outcomes^2^ of Group Positive Affect?

Twenty three studies reported outcomes of group positive affect. Although the outcomes studied were varied, we have classified them in six categories. 

*Performance*: Several authors used a measure of objective performance (e.g., solution to a problem, or a sales rate), reducing common method variance and adding robustness to the findings. For instance, Bramesfeld and Gasper [45] carried out a murder mystery task in an experimental study. In this study, the performance measure was related to a combination of suspects’ guilt ratings and the number of correct suspects. Results suggested that group positive affect has an indirect effect on group performance through the focus on the critical evidence. However, this relationship was only significant when the critical evidence was unique. Lee, Stajkovic, and Sergent [61] observed that group efficacy works as a full mediator between group positive affect and group performance (i.e., amount of metal processed each month by each group). However, group positive affect was not related to group efficacy unless low levels of group trust moderated the relationship. Another example of full mediation was found in Rego et al. [35]. Rego et al. [35] tested two proposals, finding that creativity fully mediated the relationship between group positive affect and performance (i.e., sales achievement in the current semester, or sales achievement in the subsequent semester). Moreover, negative affective tone moderates the relationship between group positive affect and performance. This relationship was found to be more intense when groups felt high levels of negative affective tone.

The aforementioned authors based their studies on different mediator mechanisms in order to explain the relationship between group positive affect and group performance. However, Knight [60] suggested a direct relation, instead of indirect. Specifically, considering team life (i.e., early, midpoint, and late), the data showed that group positive affect at the midpoint of team life was positively related to team performance (i.e., results in a competition). 

With regard to group performance evaluated by a supervisor, we found five articles that reached the same conclusion: group positive affect has a positive and significant effect on group performance [13,15,53,68,69]. However, Paulsen, et al. [67] also considered that the project phase (i.e., first and second) could influence the effect of group positive affect on team performance. The interaction analysis confirmed this influence, but it also showed that (1) the association between group positive affect and team performance was stronger in the second phase of the project than in the first phase; (2) groups that experienced high levels of positive affect displayed the same level of performance, regardless of the project phase.

On the other hand, unlike the aforementioned authors, it has been considered that the relationship is not direct, but rather mediated by the effect of the variables. Based on Broaden and Build Theory [91,92], team resilience [15] and group social resources [13] were considered as an underlying mechanism connecting group positive affect to team performance. Thus, groups that experience positive affect grow with adversity and reinforce their social bonds which allows them to complete both the required tasks and those that are not required formally by the job.

*Creativity*: Shin and colleagues [59,73,74] systematically confirmed that group positive affect would promote a collective reflection about the team´s objectives and motivate group members to actively pursue them. According to the authors, these group behaviors (i.e., team reflexivity and team promotion focus) operate as a mediating process that allows groups to achieve new solutions, but also change what does not work (i.e., organizational citizenship behavior). More recently, Shin et al. [71] suggested that transformational leadership behaviors moderate the effect of group positive affect. In fact, only when leaders exhibited high levels of transformational leadership was the indirect effect of group positive affect on team creativity via team reflexivity significant. In addition, the best levels of team reflexivity were reached when high levels of group positive affect and transformational leadership were combined. 

From a multilevel perspective, group positive affect also revealed a positive association with individual creativity. Specifically, cross-level group positive affect moderates the relationship between positive affect and creativity at the individual level. Thus, when high levels of group positive affect fit with high levels of individual positive affect, employees develop greater creativity [56]. Considered as a moderator variable of group diversity (e.g., motivations, attitudes, and professional background), high levels of group positive affect reduce the negative effects of high diversity on knowledge sharing and team creativity [73]. Finally, Tu [78] proposed those contextual factors (i.e., organizational support and organizational control) moderate the relationship between group positive affect and team creativity. Although correlations showed a positive relationship between group positive affect, team creativity, and organizational support, and group positive affect correlated negatively with organizational control, the findings do not support the initial proposal. 

*Absence*: The first studies on group positive affect began with George´s research [51] on absenteeism and prosocial behaviors. With a sample of 26 groups, regression analyses only showed that group positive affect was negatively related to absenteeism (*p* < 0.10). Several years later, Mason and Griffin [65] resumed the investigation, proposing the effect of group positive affect on group absence behavior over a one-year period. After performing several statistical analyses, the results indicated that group positive affect was negatively related to the level of group absenteeism. Moreover, the explanatory power of group positive affect improved over time. After one year, the explained between-group variance increased from 3% to 11%.

*Group efficacy*: Based on several theories (e.g., social cognitive, and broaden-and-build), different authors have provided conclusive results about the positive relationship between group positive affect and group efficacy. Specifically, group positive affect has been shown to be an antecedent of group efficacy [59,65], but also, as Salanova, Llorens and Schaufeli [16] noted in a three-wave study, the influence between these variables could be bidirectional. In other words, happy groups would develop confidence in their skills and success during the task, which would promote new positive affect among group members. Therefore, results suggest a positive spiral model. In spite of previous studies, Lee et al. [61] showed that group trust moderates the relationship between group positive affect and group efficacy. In fact, group positive affect was not related to group efficacy unless low levels of group trust moderated the relationship.

*Other group outcomes*: Tran, Paez, and Sanchez [76] established that group positive affect could be divided into two types, achievement affect (e.g., joy, satisfaction) and approach affect (e.g., interest and hope). During a decision-making task, every type of positive affect would be positive or negative for a specific main process (i.e., generation of alternatives and evaluation of alternatives). Results showed that group positive affect, such as interest and hope, was positively related to generating alternatives. On the other hand, Lin, et al. [63] tested group identification as an outcome of group positive affect, revealing that sharing positive affect among group members allows members to feel like a whole. 

*Individual wellbeing*: Belonging to a happy group may provide benefits not only for the group, but also for the members. This conclusion has been determined by several studies that verified the effect of group positive affect on individual wellbeing. For instance, group positive affect acts as a job resource that reinforces the individual’s cognition about his/her self-worth and capabilities, as well as enhancing positive group relationship precursors of individual work engagement [81]. Moreover, group positive affect could buffer individual psychological distress as the opposite of wellbeing. According to Levecque, Roose, Vanroelen, and Rossem [62], it protects against the negative effects of high job demands, reducing psychological distress. 

### 3.4. Research Question 4. Between What Variables do Group Positive Affect Works as a Psychosocial Mechanisms?

Thirteen studies reported how group positive affect worked as a mediator between several variables. We have classified the studies in three categories. 

*As mediator between leader and group outcomes*: The first study that analyzed the relationship between leadership and group outcomes was carried out by Hmieleski, Cole, and Baron [34]. The authors found that in a sample composed of top management teams, authentic leadership encourages group positive affect, which in turn, is positively related to organizational performance. Later, several studies confirmed this mediation. For example, Chi and Huang [47] tested the effect of transformational leadership on team performance by proposing a double mediation; that is, a team learning goal orientation partially mediates the relationship between transformational leadership and group positive affect, but group positive affect also fully mediates between a team learning goal orientation and team performance (i.e., Leadership→ Team learning (partial mediation) → Group positive affect (full mediation) →Team Performance). Although Sánchez-Cardona, Salanova and Llorens-Gumbau [68] also confirmed the mediating effect of group positive affect, the authors suggested a new combination in which leadership first stimulates group positive affect, which, in turn, is positively related to team learning. As Sanchez-Cardona et al. [68] noted, more studies should be conducted in order to reinforce the idea of gain spirals involving leadership, group positive affect, and group outcomes. However, research tested the effect of other types of leader characteristics, such as psychological capital. 

On the other hand, using emotional contagion as an explanatory mechanism, several authors have examined the effect of the leader´s mood on group positive affect. For instance, Chi, Chung, and Tsai [17] showed that the positive mood displayed by the leader has an effect on the group’s positive affect. SEM results indicated that group positive affect works as a mediator variable between the leader´s positive mood and team outcomes (i.e., team goal commitment, team satisfaction, and team helping behaviors). In addition, group positive affect had a significant indirect effect on team performance via these outcomes. Two subsequent studies continued with this question, adding new variables to the model. First, Volmer [83] proposed three different outcomes (i.e., team performance, potency, and goal commitment) and found that only group positive affect mediates between the leader´s mood and potency. The other two outcomes were not found to be related to group positive affect (i.e., team performance) or just showed a positive tendency (i.e., goal commitment). Second, Seong and Choi [69] confirmed the same results about the positive and significant effect of leader positive mood on group positive affect. However, the authors also observed that those groups that experience positive affect also pursue common goals, have the skills to complete the tasks, and in turn, achieve good group performance. Finally, extending the concept of emotional contagion, Zhang, et al. [81] proposed that leaders could share much more than their emotions. In fact, the authors pointed out that the leader’s psychological capital guides the development of group positive affect in their followers.

*As a mediator between group processes and group outcomes*: Support climate predicts group positive affect, and group positive affect predicts both measures of team performance (i.e., Team members’ perceived team performance and the manager’s ratings of team effectiveness) [54]. However, the relationship between support climate at Time 1 and team members’ perceived team performance at Time 3 was fully mediated by group positive affect at Time 2. On the other hand, Salanova, et al. [16], through a three-wave positive spiral model, replicated the same model at two different levels of analysis (i.e., individual, group), determining that group positive affect (i.e., enthusiasm, satisfaction, comfort) functions as a mediator variable between efficacy beliefs and engagement in a laboratory context. Finally, based on healthy and resilient organizations model [18], group positive affect has been considered a path through the group empathy could produce better quality of service [46]. That is, those groups that have social resources generate positive collective feelings that improve customer service and satisfaction of customer expectations.

*As a mediator between contextual factors and group outcomes*: The findings obtained by Dimotakis, Davison and Hollenbeck [49] were threefold. First, team structure and regulatory task characteristics had significant negative effects on group positive affect. Second, results indicated that only groups in a divisional structure and focused on gains (i.e., regulatory focus based on promotion objectives) were associated with high levels of group positive affect. Other combinations showed the lowest levels of group positive affect. Third, authors found that the moderating effect of team structure (on the relationship between regulatory focus and task satisfaction and performance) is mediated by group positive affect. However, Hentschel, Shemla, Wegge, and Kearney [55] also tested whether the interaction effect of perceived diversity and diversity beliefs had a significant influence on group positive affect. The data supported only an indirect effect of perceived diversity on identification through group positive affect. Specifically, perceived diversity was negatively associated with group positive affect, but group positive affect was positively related to identification. 

The last study included that verified the mediating effect of group positive affect was carried out by Teng and Luo [75]. In a sample of university students, they found that group positive affect had a positive and significant effect on group performance during an academic project based on group learning. However, this positive and significant effect was only confirmed for self-reported group performance, but not for objective performance measured by the professor. Moreover, the authors found that group positive affect partially mediated between social loafing and social interdependence. In fact, social loafing showed a negative effect on both group positive affect and self-reported performance, whereas social interdependence showed a positive effect on both group positive affect and self-reported performance.

### 3.5. Research Question 5. Under What Circumstances do High Levels of Group Positive Affect Lead to Negative Outcomes?

George and King [93] openly approached what they called potential pitfalls of group positive affect; that is, those circumstances where positive experiences in groups produce harmful outcomes or do not produce the expected outcomes. The pitfalls detected in the ten research studies included in the integrative review will be discussed below in three categories, depending on the related factor.

*Related to performance*: Following Social identity theory, hierarchical regression analysis revealed that when members identify with their groups, the effect of group positive affect on team performance is strengthened [74]. In fact, the effect of group positive affect alone on team performance was not significant. Thus, groups achieve the best performance when they feel high levels of group positive affect and group identification, whereas low identification levels are related to low performance (compared to high identification), regardless of the levels of group positive affect experienced. The same results were obtained for willingness to engage in OCB as an outcome. Through a laboratory study, Klep, Wisse, and Van der Flier [59] manipulated the group affect (i.e., positive and negative), as well as the affective interaction among group members, during two types of tasks (i.e., analytical and creative). The groups assigned to the positive affect condition obtained better performance on both tasks than the groups in the negative affect condition. However, the study found an exception to this rule. When groups in the positive affect condition also had the opportunity for affective interaction while performing an analytic task, they obtained the worst performance. On the analytical tasks, sharing affect kept the groups from obtaining good performance, whereas happy groups obtained the same performance on the creative task, regardless of whether they interacted and shared their affective states or not. Finally, Collins, Jordan, Lawrence, and Troth [48] developed two independent studies (i.e., study 1 and study 2) using two different laboratory tasks (decision-making and creative) in order to test how group emotional skills (i.e., management of others’ emotions) regulate the effect of group positive affect on group performance. Results indicated that the effect only makes sense when this regulation occurs. Specifically, the lowest levels of group performance occurred systematically when the group experienced high levels of positive affect but was not able to manage them, whereas the best group performance arose when the group had the ability to manage high levels of positive affect. 

*Related to group trust*: In specific situations (i.e., high levels of trust and positive affect), groups could show a tendency to undermine deviant creative ideas [77]. Moreover, Tsai et al. [80] tested a three-interaction model showing that the best team creativity was achieved when groups developed high team trust, high negative group affect, and low levels of group positive affect. However, increases in group trust could make the relationship between group positive affect and group efficacy weaker, until returning to a non-significant relationship [61]

*Related to other outcomes*: Through an experimental study using a decision-making task, Van Knippenberg, Kooij-de Bode, and van Ginkel [79] found that group positive affect could be less involved when discussing the task information and integrating it with the other members, leading them to achieve lower quality decisions than groups immersed in a negative or neutral affect. However, this would only occur when group members displayed low levels of trait negative affect. In line with these conclusions, happy groups showed lower levels of belongingness and information sharing than unhappy groups. Specifically, happy and unhappy groups showed better levels on both outcomes when members interacted and shared their affect [59]. Finally, Knight 60 related group positive affect to team exploratory search over time. Team exploratory search is understood as the intention of group members to pursue new and alternative ways to complete tasks. According to Knight’s hypothesis, group positive affect is positively related to team exploratory search during early team life, but at the midpoint of team life, group positive affect decreases team exploratory search. In fact, depending on the levels of group positive affect (i.e., high and low), the results were different. Groups with low levels of positive affect achieved higher levels of team exploratory search between early team life and the midpoint of team life, but also less descent between the midpoint of team life and late team life.

So far, literature has shown that positive affect is positively related to other positive experiences, including engagement. However, Salanova et al. [70] detected that this phenomenon did not happen in the same way with all positive affect. In fact, comfort, understood as an emotion of high pleasure and low activation, showed a negative relationship with engagement. Finally, considering the effect of positive emotions from a different perspective, Kim, Shin, and Kim [58] examined a cross-level model based on three-way interactions among group positive affect, group positive affect diversity, and individual positive affect on job satisfaction, organizational citizenship behavior, and commitment at the individual level. Data showed two results: (1) Group positive affect is positively related to job satisfaction; (2) The aforementioned three-way interaction was only positive for commitment. Plotting the results, four patterns were found (i.e., high group positive affect, high diversity; low group positive affect, low diversity; high group positive affect, low diversity; low group positive affect, and high diversity). As the authors noted, the relationship between individual positive affect and commitment was stronger when group positive affect was low, and group positive affect diversity was high.

## 4. Discussion

The purpose of the present integrative review was threefold: (1) analyze the literature in order to critically review empirical research about group positive affect, (2) synthesize the findings to more fully understand group positive affect, and (3) make proposals for future studies to advance the group positive affect research. In an attempt to logically achieve the purpose, we summarize the results following the research question addressed, as well as examine the limitations of our study, and suggest a wide research about group positive affect.

### 4.1. Research Question 1. How is Group Positive Affect Operationalized?

With regard to methodological issues in the group positive affect research; two aspects need a deeply explored. First, for studies investigating group homogeneity, response rate is a relevant data in order to determinate the validity of the group variable measure. However, there is no established requirement to represents an acceptable response rate [64]. Some authors such as Jackson et al. [94] established a response rate of 75% or higher. The present integrative review shows that 17 studies did not report the group response date, and 15 studies did not achieve this criterion. Second, about the group size, it has been observed that groups composed of more than 30 members tend to split into subgroups [95] and do not provide a representative picture of group-level effects [65]. 

On the other hand, probably the most well-known and widely used instrument in the literature is the PANAS. However, Dienet et al. [96] mentioned some limitations that may have caused some authors to decide to use another instrument. For example, PANAS assesses adjectives that are not considered emotions (e.g., determined or strong), and it measures highly activated emotions more than lowly activated ones. On the other hand, studying group positive affect from different theoretical models has produced a lack of consensus in the terminology used. In fact, the review pointed out that the variety of terms used to refer to the same construct (i.e., group positive affect) is alarming, which leads to difficulties in synthesizing the advances made in the studied construct. 

### 4.2. Research Question 2: What are the Antecedents of Group Positive Affect?

With regard to antecedents of group positive affect, a general vision suggests that the antecedents proposed so far do not seem to follow a systematic order based on clear and strong theory. Some variables have shown a positive (e.g., support climate or social interdependence) or negative (e.g., social loafing or team structure) relationship with group positive affect. On the other hand, the facilitating effect of the leader is especially remarkable. Leadership behaviors (e.g., transformational leadership), leader expression of positive affect and positive states (i.e., psychological capital) allow groups to develop higher levels of positive affect. In addition, results about the benefits of diversity and similarity in the groups are mixed. Apparently, similarity between group members (i.e., type of contract and organizational tenure) was positively related to group positive affect [53], whereas diversity (i.e., personality) has a negative relationship with group positive affect [72]. However, if other variables are considered in the model, the question is more complex. For example, absenteeism tended to be high in groups composed of a high proportion of males [65], whereas group diversity seemed to have positive effects on group performance [61], but not on creativity [73]. 

### 4.3. Research Question 3: What are the Outcomes of Group Positive Affect?

The outcomes of group positive affect seem to be wide-ranging, but clear. Group positive affect is positively related to group wellbeing (i.e., satisfaction, work engagement, and group efficacy, potency), group processes (i.e., identification and team learning), group performance, creative performance, other outcomes (i.e., help behaviors, commitment, skills, and pursue goals), and individual wellbeing. Furthermore, group positive affect showed a negative relationship with absence. Specifically, for researchers there has always been a growing interest in relating positive affect to performance. As far as we know, this relationship has commonly been called the happy-productive worker [97], and it has been analyzed from multiple perspectives and areas [98,99]. Based on happy-productive worker research and group theories, some authors have started a new research about happy-productive group [13,22] with promising results. 

On the other hand, considering theories such as Broaden and Build Theory [91,92], it is plausible to consider that high levels of positive affect do not automatically imply high levels of performance, but instead the mediating effect would cause this to occur. At the group level of analysis, Kelly and Spoor [100] stated that few studies have addressed mechanisms that could explain the aforementioned relationship. Supporting the previous statement, the present review found that only eleven studies linked group positive affect to group performance, proposing three different types of mechanisms: cognitive mechanisms (e.g., group efficacy), behavioral mechanisms (e.g., team resilience), and external mechanisms (e.g., phase project).

### 4.4. Research Question 4: Between What Variables do Group Positive Affect Works as a Psychosocial Mechanisms?

To analyze group positive affect addressing what causes it or the effects it has, leads to a diffuse and restrictive picture of the construct. Various studies understand group positive affect as a mechanism that connects different variables with each other. Data show that the most studied relationship is between leader and group outcomes through group positive affect. Following Job Demands-Resources Theory [101], it is plausible that the leader connects with the group through collective well-being (i.e., group positive affect) so that the group could enhance the outcomes. Although the other studies also suggest group positive affect as a mechanism for achieving group outcomes, they are based on very dissimilar theories. 

### 4.5. Research Question 5: Under What Circumstances do High Levels of Group Positive Affect Lead to Negative Outcomes?

Several authors have suggested different circumstances where the completely advantageous effects of group positive affect have been questioned. For example, group and individual outcomes (e.g., performance, creativity, quality decision, team exploratory search, and individual commitment) could be reduced depending on whether the members identify with their group, or depending on emotional competences, interaction during the task, the moment in the team life, affective diversity, the type of task (i.e., creative or analytical), and negative affect (i.e., individual or group). After an analysis of the pitfall research, and without undermining previous research, we have become aware that (1) There are studies where the task performed by the groups was evaluated with a scale that did not capture the true value of the performance. For instance, a creative task should be measured using criteria for creative performance and not task performance. (2) The pitfalls focus on what happens when groups exhibit high levels of positive affect and low results, but we do not know what happens when positive affect is low and good results are obtained. (3) The circumstances in which group positive affect produces negative effects are quite varied and complex. However, it is necessary to establish which differences allow the groups to obtain good results. 

### 4.6. Implications for Practice

In terms of practical implications, our research promotes several empirically-based human resources strategies related to recruitment, group design and leadership training, in order to promote group positive affect. 

First, recruitment is the first step in choosing employees who would fit well within a group. As noted by Gil, Llorens, and Torrente [53], when group members perceive themselves as equals and have a sense of affiliation with the group, they could develop a shared group positive affect. 

With regard to group design, Job Demands-Resources Theory [101] identifies job resources and job demands as a wide range of work characteristics significantly related to employee wellbeing at individual, but also, at group level [102]. Organizations concerned with collective wellbeing (i.e., group positive affect) could invest energy and resources to assess team characteristics. Further, Oldham and Hackman [103] mentioned that some characteristics may be considered, such as type of task and type of group.

Finally, the present review has shown that the leader plays a key role in developing group positive affect through different mechanisms (e.g., leadership style, personality, leader attraction, and emotional contagion susceptibility). In fact, there is a substantial body of research on the meaning of leadership for employee and group wellbeing, as well as for organizational outcomes [104]. For instance, Kelloway and Barling [105], after analyzing several interventions based on leadership training, concluded that (1) interventions in leadership produce improvements in the leader him/herself and not only in his/her followers; (2) the interventions should not only be focused on the immediate supervisor, but also on mid-level and high-level managers because, due to a cascade effect, the lower levels could benefit from this intervention, or it could even have different effects on the employee. Therefore, leadership training could be incorporated into organizational practices.

### 4.7. Limitations 

There are a few limitations associated with this study.

First, we are aware that restricting the search to published scientific articles could lead to publication bias [106]. However, despite the use of professional social networks (i.e., research gate) and scientific databases to obtain information, it is often difficult for researchers to access unpublished studies.

Second, as Menges and Kilduff [9] noted, researchers have used a wide variety of terms to refer to positive affective experiences in groups, causing great difficulties in selecting keywords during the search strategy. In order to minimize this difficulty, a manual search was carried out that complements the limitations of searching through key words in titles and abstracts.

Third, although several of the articles analyzed mentioned group positive affect e.g., [107,108], they were excluded because the aggregation indices (i.e., agreement and reliability) were not performed. These analyses allow us to statistically assume that group positive affect is shared among group members. However, not including these articles might mean that the full scope of group positive affect was not reached. 

Finally, in spite of number of articles analyzed, it has not been possible to develop a meta-analysis because the literature on group positive affect is diverse and unstructured. In fact, to date, only one meta-analysis has been carried out [23]. This meta-analysis aimed to relate group positive affect to social integration, and group performance.

### 4.8. Future research Agenda

As a result of the present review, below we discuss four topics that seem highly relevant for further progress in group positive affect research.

*A multilevel approach about the antecedents and consequences of positive affect*: Although groups and organizations are multilevel structures that require a multilevel approach [109], most previous studies on group positive affect have focused on analyzing the construct at the group level of analysis, leaving out cross-level effects. For example, currently, little knowledge is available about the effect of group positive affect on individual well-being (e.g., work engagement) or even on individual behaviors such as job crafting [110]. On the other hand, it has been shown that the outcomes of group positive affect have been considered more relevant than their drivers. Following HERO Model [39], organizational strategies might boost different collective wellbeing such as group positive affect. Further research could investigate a multilevel approach that simultaneously takes into consideration the different levels of positive affect in organizations (i.e., individual, group, and organizational) would be essential to establish whether there are relationships between them, as well as possible effects and cross-level relationships with other variables. 

*Diversity in the organizations*: Due to current social changes, it is essential for organizations to manage diversity in their teams [111]. Recently, it has been demonstrated the implications of diversity for organizations. For instance, Curşeu, Pluut, Boroş, and Meslec [112] showed that groups composed by high percentage of women promote the emergence of group emotional intelligence, which in turn reduces relationship conflict and increases group cohesion. Through a laboratory study, Martínez and Cifre [113] reached similar conclusions about gender diversity. The gender diversity negatively modulates the relationship between self-efficacy and satisfaction. That is, homogeneous groups (i.e., gender) with high levels of self-efficacy achieve higher levels of satisfaction. Taking into account the negative effects of diversity on group positive affect, researchers might explore how match organizational diversity with desired results.

*Happy-unproductive or unhappy-productive?* Recently, Peiró, Kozusznik, Rodríguez-Molina, and Tordera [114] noted that the relationship between positive affect and performance is more complex than the happy-productive thesis proposes. In fact, the authors found a relationship with four patterns. That is, employees might be happy-productive, happy-unproductive, unhappy-productive, or unhappy-unproductive. At group level, George and King [93] hypothesized a rationale behind happy-unproductive pattern. George and King [93] noted that positive states experienced in a group strengthen each other by building a shared reality that might intensify group conformity, but also a false perception of reliable, when the circumstances indicate the opposite. In other words, groups might discourage misaligned ideas and seek complacency, leading to poor performance. Literature shed light on what circumstances group positive affect boost harmful outcomes such as poor management of others’ emotions and high levels of group trust. Assuming that groups are social systems with emergent properties [93], further research could explore the existence of the four group patterns (i.e., happy-productive, happy-unproductive, unhappy-productive, or unhappy-unproductive), as well as the conditions that lead the groups to belong to them.

*Affective dynamics*: Considering the importance of time for groups and teams [115], it would be interesting to examine the changes over time in the relationship between group positive affect and the related variables (i.e., antecedents, outcome, mediators, and moderators). With the exception of Salanova et al. [16], we lack results about possible feedback between group positive affect and the variables related to it. For example, is there feedback between group positive affect and positive outcomes (e.g., group performance and group commitment), so that group positive affect enhances positive outcomes, which, in turn, develop group positive affect? Is there a moment when group positive affect does not influence teams, depending on their team life? Moreover, based on Broaden and Build Theory [91,92], how long would it take for team resources to be generated by group positive affect? 

## 5. Conclusions

Finding suggests that scholars have been more interested in understanding the outcomes of group positive affect and how to improve the productivity of groups than in knowing what the antecedents are. A summary conclusion is that group positive affect is related to leadership, job demands, job resources, diversity/similarity, group processes, and contextual factors, all of which influence the development of several outcomes and different types of wellbeing at the individual and group levels. However, with specific combinations of other conditions (e.g., group trust, negative affect, and interaction), high levels of group positive affect could cause harmful results. The present integrative review proposes a wide future research agenda, suggesting the study of antecedents and consequences of group positive affect from a multilevel approach, the effect of organizational diversity on group positive affect, anomalous group pattern (e.g., happy-unproductive) and affective dynamics. Conclusions shed light on group positive affect research and practice and might help Human Resources professionals to initiate empirically-based strategies related to recruitment, group design and leadership training.
